# Caregiving History’s Associations to Later-Life Social Disconnectedness and Loneliness

**DOI:** 10.1093/geroni/igaf122.1861

**Published:** 2025-12-31

**Authors:** Rita Hu, Ji Hyun Lee

**Affiliations:** The University of Chicago, Chicago, Illinois, United States; Montana State University, Bozeman, Montana, United States

## Abstract

Throughout adulthood, many people provide care for loved ones. While research has documented both the stressors and rewards of caregiving, less is known about caregiving experiences over the life course and their long-term implications for later-life social well-being. This study examines whether earlier-life caregiving experiences predict later-life social disconnectedness and loneliness. Using the Health and Retirement Study (HRS)’s retrospective Life History Mail Survey (2015/2017/2019; N = 6,373), we identified individuals who had ever provided unpaid care for six or more months to people with chronic conditions. Social disconnectedness (indexed by close network size, network diversity, contact frequency, and social participation) and loneliness (11-item UCLA scale) were measured in the 2016/2018 HRS Psychosocial Leave-Behind Questionnaire. Covariates included later-life material status, memory, and physical and mental health. Results showed that individuals with any caregiving history (vs. non-caregivers) were less likely to experience social disconnectedness (β = -0.08, p < .001) and general loneliness (β = -0.02, p = .04) in later life. Further analysis revealed that caregiving history was associated with higher social participation (β = -0.07, p < .001) but not with close network size, network diversity, or contact frequency. For loneliness, caregiving predicted lower collective loneliness (β = -0.06, p < .001), with stronger effects for men. Stratified analyses showed that both genders benefited from lower collective loneliness; caregiving was also associated with lower social loneliness among men only (β = -0.044, p = .002). Findings highlight the enduring positive social health outcomes of earlier-life caregiving, particularly for men.

